# De-escalating first-line treatment in stage IVB or recurrent cervical cancer: outcomes of immunotherapy alone and systemic review

**DOI:** 10.1093/oncolo/oyaf096

**Published:** 2025-05-27

**Authors:** Akram Saad, Alexandra Taylor, Shira Felder, Limor Helpman, Smadar Bauer, Ronnie Shapira, Keren Levanon, Jacob Korach, Ronza Atamneh, Samantha Breslauer, Jeffrey Goldstein, Shira Peleg Hasson

**Affiliations:** Faculty of Medicine, Tel Aviv University, P.O.B 39040 Ramat Aviv, Tel Aviv 69978, Israel; Sheba Cancer Center and Institute of Oncology, Tel-Hashomer, Derech Sheba 2, Ramat Gan, Israel; The Royal Marsden, Department of Clinical Oncology, 203 Fulham Rd., London SW3 6JJ, United Kingdom; Faculty of Medicine, Tel Aviv University, P.O.B 39040 Ramat Aviv, Tel Aviv 69978, Israel; Sheba Cancer Center and Institute of Oncology, Tel-Hashomer, Derech Sheba 2, Ramat Gan, Israel; Faculty of Medicine, Tel Aviv University, P.O.B 39040 Ramat Aviv, Tel Aviv 69978, Israel; Gynecologic Oncology, Sheba Medical Center, Tel-Hashomer, Derech Sheba 2, Ramat Gan, Israel; Faculty of Medicine, Tel Aviv University, P.O.B 39040 Ramat Aviv, Tel Aviv 69978, Israel; Sheba Cancer Center and Institute of Oncology, Tel-Hashomer, Derech Sheba 2, Ramat Gan, Israel; Faculty of Medicine, Tel Aviv University, P.O.B 39040 Ramat Aviv, Tel Aviv 69978, Israel; Sheba Cancer Center and Institute of Oncology, Tel-Hashomer, Derech Sheba 2, Ramat Gan, Israel; Faculty of Medicine, Tel Aviv University, P.O.B 39040 Ramat Aviv, Tel Aviv 69978, Israel; Sheba Cancer Center and Institute of Oncology, Tel-Hashomer, Derech Sheba 2, Ramat Gan, Israel; Faculty of Medicine, Tel Aviv University, P.O.B 39040 Ramat Aviv, Tel Aviv 69978, Israel; Gynecologic Oncology, Sheba Medical Center, Tel-Hashomer, Derech Sheba 2, Ramat Gan, Israel; Faculty of Medicine, Tel Aviv University, P.O.B 39040 Ramat Aviv, Tel Aviv 69978, Israel; Rappaport Faculty of Medicine, Technion Israel Institute of Technology, 1 Efron St. Bat Galim, Haifa 3525433, Israel; Tel Aviv Sourasky Medical Center, Department of Radiation Oncology, Weizmann St 6, Tel Aviv-Yafo, Israel; Faculty of Medicine, Tel Aviv University, P.O.B 39040 Ramat Aviv, Tel Aviv 69978, Israel; Tel Aviv Sourasky Medical Center, Department of Medical Oncology, Weizmann St 6, Tel Aviv-Yafo, Israel

**Keywords:** cervical cancer, immunotherapy, stage-IVB, metastatic, radiation, survival

## Abstract

**Introduction:**

Chemo-immunotherapy (IO) is the preferred first-line treatment for stage IVB or recurrent cervical cancer. However, limited data exist on the efficacy and safety of using IO-alone as a de-escalation strategy. We report outcomes from a case series of selected patients treated with IO-alone and review the feasibility of de-escalating first-line treatment.

**Methods:**

The authors conducted a literature review using Google Scholar and PubMed to identify reports using IO-alone as a de-escalation strategy across malignancies published between 1999 and December 2024 and also reviewed a cervical cancer database from a tertiary academic to identify patients with stage IVB or recurrent disease treated with IO-alone. The authors used the Kaplan-Meier method to estimate progression-free survival (PFS) and overall survival (OS).

**Results:**

Among 582 patients treated between 2015 and 2021, 18 met the inclusion criteria. The median age was 43 years (range 28-84); 67% had squamous cell carcinoma, 11% adenocarcinoma, and 80% expressed PD-L1. CPS scores were <1 in 20%, 1--10 in 33%, and >10 in 47%. Most patients had oligo-metastatic disease (83%). Treatment with IO-alone began a median of 7 months after platinum-based chemotherapy. Indications included prior adjuvant (44%) or neoadjuvant (22%) chemotherapy, clinical trial participation (11%), or patient preference (22%). Median PFS and OS were 27 months and 82 months, respectively.

**Conclusions:**

These findings support the need for clinical trials evaluating IO-alone as a first-line treatment option for de-escalation in stage IVB or recurrent cervical cancer. Biomarker development is needed to better identify candidates for personalized therapy.

**Implications for practice**

Chemo-IO remains the preferred first-line treatment for patients with stage IVB or recurrent cervical cancer. However, in other malignancies, such as lung and bladder cancer, biomarker-driven treatment strategies have validated the use of IO-alone. In this study, we describe a cohort of patients who received treatment with IO-alone as a de-escalating strategy. These findings highlight the need for further investigations into de-escalated palliative therapy in this patient population to refine treatment options and improve patient outcomes.

## Introduction and review of current literature

Cervical cancer, a preventable and treatable disease, ranks as the fourth most common cancer and the fourth leading cause of cancer-related deaths among women.^1^ While few women present with metastatic disease at initial diagnosis (~10%),^1,2^ recurrence occurs in 15%-61% of women within the first 2 years following primary treatment.^3^ Historically, patients with recurrent or metastatic cervical cancer ineligible for curative interventions with surgery or radiation therapy (RT) received platinum-based chemotherapy with bevacizumab.^4,5^ This combination achieves a median progression-free survival (PFS) of 8.2 months and overall survival (OS) of 17 months.^5^ These outcomes underscore the need for novel strategies to improve survival while minimizing treatment-associated toxicity.

The integration of immunotherapy (IO) into the treatment paradigm has expanded the treatment options for patients with cervical cancer. The identification of biomarkers such as tumor mutational burden (TMB),^6^ microsatellite instability (MSI),^7^ and programmed death-ligand 1 (PD-L1) expression,^8^ highlights the immunogenic potential of cervical cancer and the rationale for IO-based approaches. Furthermore, RT-induced immunomodulation may amplify the response to IO by enhancing antigen presentation, T-cell activation, and pro-inflammatory cytokine release.^9^ Promising results from early IO trials such as Keynote-158^10^ and Checkmate-358^11,12^ in patients with metastatic or recurrent cervical cancer resulted in FDA approval of pembrolizumab as a second-line therapy for patients with cervical cancer whose tumors express PD-L1 (CPS ≥ 1).^12^ The pivotal phase-III Keynote-826 trial established the addition of pembrolizumab to chemotherapy (±bevacizumab) as a first-line agent in patients with recurrent or metastatic cervical cancer,^13^ improving the 2-year OS rate to 50.4% from 40.4%.

Subgroup analysis demonstrated favorable outcomes for most groups except those with low PD-L1 combined positive scores,^13^ underscoring the importance of biomarker-based therapies. In 2021, the combination of pembrolizumab with chemotherapy±bevacizumab gained approval as a first-line treatment for PD-L1-positive, persistent, or recurrent/metastatic cervical cancer.^14^

IO-alone has emerged as a transformative approach in the first-line treatment of various metastatic malignancies. In non-small cell lung cancer (NSCLC), pembrolizumab has become a standard first-line therapy for patients with high PD-L1 expression (≥50%) and no EGFR or ALK mutations, demonstrating improved OS compared to chemotherapy alone.^15^ Similarly, in metastatic colorectal cancer (CRC), pembrolizumab is indicated for MSI-high or mismatch repair deficient (dMMR) tumors. Pembrolizumab demonstrated significantly superior PFS compared to standard chemotherapy in this biomarker-defined subset.^16^ These examples underscore biomarkers’ role in guiding the application of IO-alone in the first-line setting.

Beyond biomarker-driven indications, IO-alone also serves as an alternative to chemotherapy in populations with limited treatment options. In metastatic urothelial carcinoma, atezolizumab is approved for patients with PD-L1-positive tumors ineligible for cisplatin-based chemotherapy, providing an effective and well-tolerated option in a typically frail population.^17^ Furthermore, in advanced melanoma, IOs such as pembrolizumab and nivolumab have become foundational first-line treatments, achieving durable responses regardless of PD-L1 status.^18^ These studies collectively highlight the adaptability of IO-alone across malignancies, particularly in settings where either biomarker profiles predict robust responses or patient factors preclude the use of standard chemotherapy. Such advancements continue to redefine the therapeutic landscape across different malignancies, enabling precision medicine approaches tailored to individual patient and tumor characteristics.

De-escalating first-line therapy in selected patients with recurrent or metastatic cervical cancer to IO-alone instead of chemo-IO may reduce treatment toxicity and enhance quality of life. This strategy, which has already demonstrated efficacy in other malignancies,^19^ has the potential to avoid toxicities associated with more aggressive treatments. However, in the absence of phase-III trials that address this question, real-world data becomes critical to guide clinical decision-making. This review synthesizes real-world data from a case series with current evidence for using IO-alone as a first-line treatment, evaluates its impact on prognosis and toxicity, and highlights areas for further investigation. By examining patients’ characteristics and outcomes measured by PFS, OS, and treatment toxicity, we seek to contribute insights into optimizing tailored treatment approaches and improving outcomes in this challenging clinical situation. This data will serve as a basis for a future clinical trial investigating the option of using IO-alone as an alternative to chemo-IO in patients with stage IVB or recurrent cervical cancer.

## Methods

### Literature review

We conducted a systematic search in Google Scholar and PubMed databases to evaluate the use of IO-alone in cervical cancer using the following keywords: immunotherapy, immune checkpoint inhibitors, checkpoint inhibitors, PD-1, PDL1, CTLA-4, anti-PD-1 therapy, anti-PD-L1 therapy, anti-CTLA-4 therapy, nivolumab, pembrolizumab, ipilimumab, avelumab, durvalumab, atezolizumab, and monotherapy.

### Patient population of case series

The authors conducted a retrospective study using a cervical cancer database encompassing patients treated at Sheba Cancer Center between June 2015 and March 2023. Eligibility criteria included patients aged≥18 with biopsy-confirmed uterine cervix cancer, stage IB_1_-IVB, according to Federation International of Gynecology and Obstetrics (FIGO) criteria (2018).^20^ All patients included in the case series received local treatment to the cervix with radiation therapy or surgery. Exclusion criteria included patients aged <18 years, follow-up <90 days, patients lost to follow-up, patients who did not receive treatment for local disease, and patients with cancers originating from sites other than the cervix (eg, vulvar or vaginal cancer).

### Variables collected

Data extracted from the electronic medical record for analysis included patient age and stage at diagnosis, tumor histology, programmed death ligand one (PD-L1) status, combined pathological score (CPS)—defined as the total number of tumor cells, lymphocytes, and macrophages divided by the number of viable tumor cells × 100, lymph node involvement, and sites of metastatic disease sites. We grouped PD-L1 CPS values for analyses as negative or positive, or < 1%, 1%-10%, or >10%.

### Treatment parameters

Treatment data encompassed the primary treatment and therapy for recurrence, including the time intervals between initial treatment, recurrence, and initiation of IO-alone.

### Treatment of oligo-metastatic disease

Stage IVB patients may have received RT to metastatic sites concurrently with pelvic RT or at the time of oligo-metastatic recurrence, achieved by modifying pelvic RT fields, adding additional RT treatment fields, and employing techniques such as stereotactic body RT (SBRT) or surgical resection.^21^ Radiation therapy adhered to department protocols^21^ and consensus guidelines,^22,23^ with all doses standardized to biological equivalent doses (BED) using an alpha/beta ratio of 10.^24^ A prior publication details RT dosages, fractionation, and techniques used in this patient cohort.^21^ Patients received platinum therapy concomitantly with RT.

#### Treatment of oligo-metastatic or recurrent disease with IO-alone

Patients treated with IO-alone participated in clinical trials, received recent neoadjuvant or adjuvant chemotherapy, or refused chemotherapy. According to the Moore criteria,^25^ patients with intermediate or high-risk disease received bevacizumab.

### Statistical analysis

The investigators used descriptive statistics and the Kaplan-Meier (KM) method to estimate PFS from the date of initiating systemic therapy and OS from the date of diagnosis. Calculations encompassed median PFS and OS, hazard ratios (HRs) with 95% confidence intervals (CIs), and *P*-values. All statistical analyses utilized a significance threshold of *P* ≤ .05. The investigators used IBM SPSS 25.0 software (SPSS Inc.) for all statistical analyses.

### Study ethics

The institutional ethics committee (IRB) at Sheba Medical Center approved this study under Identifier SMC-5899-19. The retrospective nature of the study justified waiving informed consent.

## Case series

### Patient population

**Figure 1.**
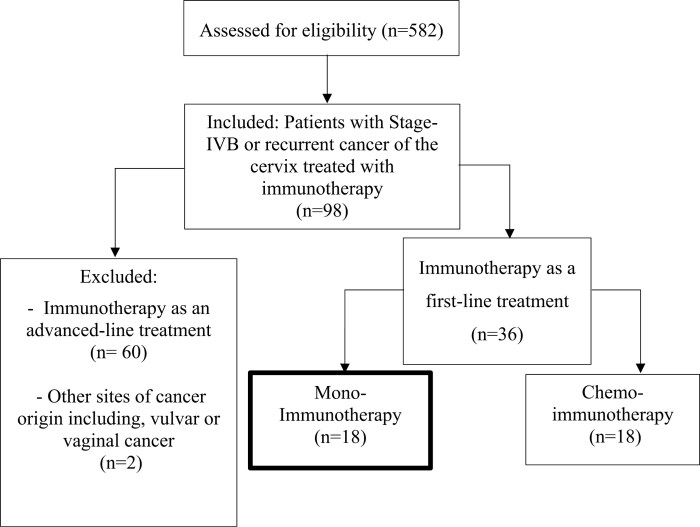
CONSORT diagram.

A review of the database (*n* = 583) identified 18 patients treated with first-line IO-only who were eligible for inclusion in the study (Figure 1). Table 1 shows the median age at diagnosis was 43 years (range: 28-84, 95% CI, 40-56). The majority of patients presented with an advanced FIGO stage: 33% IIIC1 (95% CI, 14-59%), 28% IIIC2 (95% CI, 11-54%), and 28% IVB (95% CI, 11-54%). Earlier stages, such as IB1 and IIB, were less common, with each accounting for 5.6% of cases (95% CI, 0.29-29%). Oligo-metastatic disease was prevalent in 83% of cases (95% CI, 58-96%), while 17% had poly-metastatic disease (95% CI, 4.4-42%).

**Table 1:** Patient characteristics.

Number of patients, female			IO only, *N* = 18
Age		43 (28,84)	median (range)
			95% CI
Stage at diagnosis	IB1	1 (5.6%)	0.29%, 29%
	IIA1	0 (0%)	0.00%, 22%
	IIB	1 (5.6%)	0.29%, 29%
	IIIC1	6 (33%)	14%, 59%
	IIIC2	5 (28%)	11%, 54%
	IVA	0 (0%)	0.00%, 22%
	IVB	5 (28%)	11%, 54%
Histologic subtypes	Squamous cell carcinoma	12 (67%)	41%, 86%
	Adenocarcinoma	2 (11%)	1.9%, 36%
	Other	4 (22%)	7.4%, 48%
PDL1 CPS status	< 1	3 (20%)	5.3%, 49%
	≥ 1-9%	5 (33%)	13%, 61%
	≥ 10%	7 (47%)	22%, 73%
Median time (months) from end of platinum chemotherapy to start of IO		7 (2,53)	5.6, 20
First pattern of failure	Distant recurrence	10 (56%)	31%, 78%
	Radiation filed recurrence	4 (22%)	7.4%, 48%
	Regional recurrence	4 (22%)	7.4%, 48%
Rational for IO only treatment	Adjuvant chemotherapy	8 (44%)	22%, 69%
	Participation in clinical trial Neoadjuvant chemotherapy	2 (11%) 4 (22%)	1.9%, 36% 7.4%, 48%
	Patient preference	4 (22%)	7.4%, 48%

Abbreviations: IO- immunotherapy, CI- confidence interval.

#### Tumor characteristics

Table 1 also shows that 67% of patients had squamous cell carcinoma (95% CI, 41-86%), followed by 11% with adenocarcinoma (95% CI, 1.9-36%) and 22% classified as “other” histology (95% CI, 7.4-48%). Evaluation of PD-L1 CPS status showed 20% had a CPS < 1 (95% CI, 5.3-49%), 33% had a CPS between 1-10% (95% CI, 13-61%), and 47% had CPS > 10% (95% CI, 22-73%).

#### Treatment timing and patterns of failure

The median time from the end of platinum-based therapy to initiating IO-alone was 7 months (range: 2-53, 95% CI, 5.6-20). The most common pattern of failure following initial treatment was distant recurrence in 56% of cases (95% CI, 31-78%), followed by radiation field and regional recurrence, each observed in 22% of patients (95% CI, 7.4-48%).

#### Reasons for choosing IO-alone

Reasons for selecting IO-alone as the sole treatment modality included either prior treatment with adjuvant or neoadjuvant chemotherapy (44%, 95% CI, 22-69%) and (22%, 95% CI, 7.4-48%), respectively, participation in a clinical trial (11%, 95% CI, 1.9-36%), or patient preference (22%, 95% CI, 7.4-48%).

#### Patient outcomes

**Figure 2.**
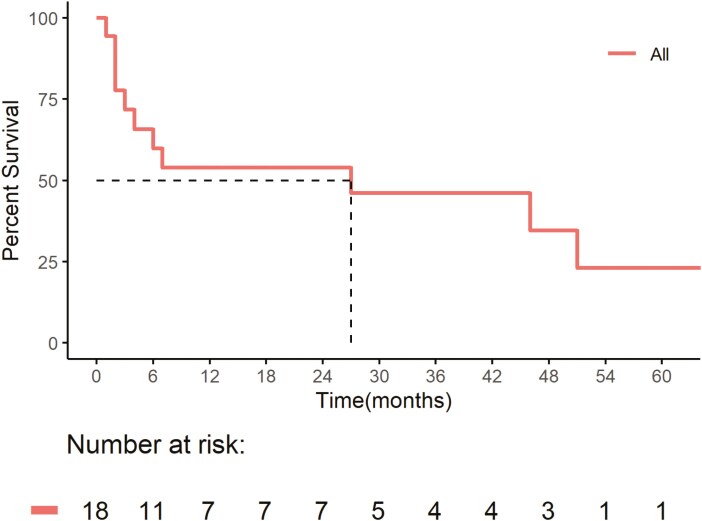
Progression-free survival of immunotherapy-alone. Progression-free survival for the entire cohort (*n* = 18) showed a median PFS of 27 months (95% CI, 4—not reached (NR)).

**Figure 3.**
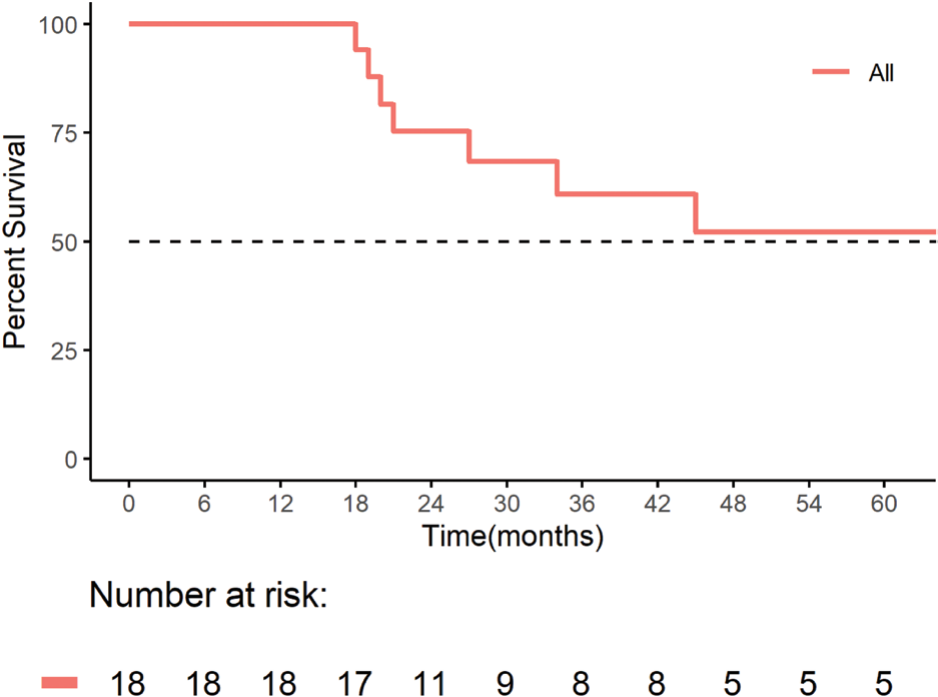
Overall survival of immunotherapy-alone. Overall survival for the entire cohort (*n* = 18) showed a median OS of 82 months (95% CI, 27-NR).

At the time of analysis, 22% of patients (95% CI, 7.4-48%) had completed 2 years of IO-alone, while 11% (95% CI, 1.9-36%) discontinued due to IO-related adverse events (iAE). Another 22% had ongoing treatment (95% CI, 7.4-48%), and 44% had discontinued due to disease progression (95% CI, 22-69%). The median duration of response to IO-alone was 8 months (range: 2-78, 95% CI, 8.7-31), and the median number of IO cycles completed was 10 (range: 2-59, 95% CI, 9.0-28). Figures 2 and 3 show Kaplan-Meier survival estimates for the entire cohort (*n* = 18) showed a median PFS of 27 months (95% CI 4—not reached (NR)) and a median OS of 82 months (95% CI, 27-NR).

## Discussion

### Summary of main results

In this series, 83% of cases had oligo-metastatic disease, while 17% had poly-metastatic disease. The median time from the completion of platinum-based therapy to the start of IO-alone was 7 months (range 2-53). Indications for treatment with IO-alone included previous adjuvant chemotherapy (44%), participation in clinical trials (11%), neoadjuvant chemotherapy (22%), and patient preference (22%). The median PFS and OS were 27 and 82 months, respectively.

The Checkmate-358^11,12^ and Keynote-158^10^ trials demonstrated the efficacy of IO-alone in the management of patients with recurrent or metastatic cervical cancer. These trials assessed nivolumab and pembrolizumab monotherapy in cervical cancer patients with recurrent or metastatic disease that progressed after first-line chemotherapy and reported median PFS and OS durations for nivolumab of 5.1 months and 21.9 months, respectively, and for pembrolizumab, 4.1 months and 23.5 months, respectively. These promising results using IO-alone in second and advanced-line settings after patients progressed on first-line chemotherapy prompted the development of phase-III trials investigating IO + chemotherapy in first-line settings.

Although no randomized control trials compare IO-alone to chemo-alone or chemo-IO in the first-line setting, the phase III Keynote-826 (NCT03635567),^13^ BEATcc (NCT03556839),^26^ and Fermata (NCT03912415)^27^ trials compare chemo-IO with chemo-alone as a first-line systemic treatment in patients with recurrent, persistent, or metastatic cervical cancer. Key findings from the Keynote-826 trial, which assessed the addition of pembrolizumab to chemotherapy (± bevacizumab), showed a median PFS and OS of 10.4 and 26.4 months, respectively for the chemo-IO group compared to 8.2 and 16.8 months, respectively for chemo-alone.^28^ The BEATcc trial showed at the interim survival analysis^29^ that the addition of atezolizumab to chemotherapy and bevacizumab improved the median PFS and OS (13·7 and 32.1 months, respectively) compared to PFS and OS with standard therapy (10·4 and 22.8 months, respectively). Our case series describing first-line systemic treatment with IO-alone compares well with these outcomes (median PFS and OS of 27 and 84 months, respectively). These findings suggest a need for additional trials evaluating IO-alone as an alternative to chemo-IO for first-line treatment of selected patients with recurrent or metastatic cervical cancer.

### Strengths and weaknesses

Strengths of this study encompass extensive data collection, thorough detailed clinical, pathological, and treatment analysis parameter reviews, and long-term outcome tracking of patients treated with novel agents in a tertiary medical center. However, the small cohort size limits the statistical power of this study, precluding conclusions about the impact of treating with IO-alone on PFS or OS. While retrospective studies inherently carry selection biases, potentially favoring more aggressive treatment for patients with better performance status and more favorable prognostic variables, our the performance status and prognostic variables in our cohort matched well with the patients treated with IO + chemotherapy at our institution.

### Implication for practice and future research

As the treatment landscape for advanced cervical carcinoma evolves, novel strategies to de-escalate therapeutic intensity warrant exploration. Initiating a phase-3 clinical trial comparing outcomes with IO-alone and chemo-IO as first-line therapy for patients with persistent, recurrent, or metastatic cervical cancer may help direct the selection of optimal therapeutic choices. As we aim to improve patient outcomes and reduce treatment toxicities, further investigation of tailored approaches for patients receiving palliative care becomes paramount.

## Data Availability

The data supporting the findings of this review and case series are available from the corresponding author upon reasonable request.
